# Hyperhomocysteinemia as a Common Denominator: Linking Posterior Subcapsular Cataract and Pulmonary Thromboembolism

**DOI:** 10.7759/cureus.107913

**Published:** 2026-04-28

**Authors:** Atishay Jain, Varsha Luthra, Ritika Gupta, Anuraj Kaushik

**Affiliations:** 1 Internal Medicine, Government Medical College and Hospital, Chandigarh, IND; 2 General Medicine, Government Medical College and Hospital, Chandigarh, IND; 3 Radiodiagnosis, Government Medical College and Hospital, Chandigarh, IND

**Keywords:** hyperhomocysteinemia (hhcy), ocular manifestations, posterior subcapsular cataract, pulmonary embolism (pe), thrombophilia

## Abstract

Hyperhomocysteinemia (HHcy) is a condition that can lead to serious blood clotting and vascular problems. However, its effects on the eyes are less well characterized. A link between high homocysteine levels and the formation of cataracts, especially posterior subcapsular cataracts, has been established. It’s thought to be caused by damage to the lens proteins due to oxidative stress. We encountered a 40-year-old man who had a severe pulmonary embolism and was experiencing vision problems. We found that he had bilateral cataracts and high homocysteine levels (98 µmol/L). Despite testing for other conditions that could cause thromboembolism, no alternative etiology was identified after extensive evaluation. This led us to believe that his high homocysteine levels were likely the underlying cause. He was treated with thrombolytics, anticoagulants, and vitamins to lower his homocysteine levels. He was also referred for cataract surgery. This case shows how important it is to consider HHcy in patients who have cataracts at a young age, also highlighting its role as a systemic problem affecting both the blood vessels and the eyes.

## Introduction

Hyperhomocysteinemia (HHcy) is a metabolic disorder characterized by elevated plasma levels of homocysteine, which is a sulfur-containing amino acid made in methionine metabolism. Homocysteine is normally metabolized through remethylation to methionine. This process normally needs folate, vitamin B12, and trans-sulfuration to cysteine, which needs vitamin B6. Disruption of these metabolic pathways due to nutritional deficiencies, renal dysfunction, or genetic alterations like methylenetetrahydrofolate reductase (MTHFR) mutations results in the accumulation of homocysteine in circulation [1, 2]. Elevated homocysteine levels have been widely implicated in endothelial dysfunction, oxidative stress, and activation of prothrombotic pathways. This makes HHcy an established risk factor for cardiovascular and thromboembolic disease [3].

Beyond systemic vascular complications, HHcy has also been increasingly associated with ocular disorders. This included conditions like retinal vascular occlusions, optic neuropathy, glaucoma, and cataract formation [4]. Cataract development in this setting is presumed to occur due to homocysteine-induced oxidative stress and disruption of lens protein homeostasis, which leads to the structural modification and aggregation of crystallin proteins [5, 6]. Thus, these mechanisms may predispose individuals to early-onset cataracts, which are commonly associated with metabolic disturbances [7].

## Case presentation

A 40-year-old man presented with acute-onset dyspnea progressing from modified Medical Research Council (mMRC) grades I-IV over five days, accompanied by orthopnea and right-sided pleuritic chest pain, which worsened on inspiration for three days. He reported a single undocumented febrile episode five days prior without any history of palpitations, paroxysmal nocturnal dyspnea, pedal edema, decreased urine output, weight loss, diabetes, or hypertension.

He also complained of progressive bilateral blurring of vision for six weeks, which was more pronounced in bright light and while reading, and was associated with difficulty with near vision, glare, nyctalopia, and a reduction in visual acuity. There was no history of ocular trauma, chronic uveitis, intraocular surgery, prolonged use of systemic or topical corticosteroids, radiation exposure, excessive ultraviolet exposure, smoking, alcohol abuse, or use of medications known to predispose to cataract formation. There was no personal or family history of early-onset cataract, congenital ocular disorders, or connective tissue disease. He denied symptoms suggestive of diabetes mellitus, hypocalcemia, hypothyroidism, chronic kidney disease, chronic liver disease, or malabsorption, and there was no history of prolonged diarrhea, bariatric or gastrointestinal surgery, or nutritional deficiency states.

On examination, the patient was tachycardic (118 beats/min), tachypneic, hypotensive (88/58 mmHg), and hypoxic with an SpO₂ of 80% on room air. Chest auscultation revealed a left-sided wheeze. Other systemic examinations were unremarkable. Initial laboratory evaluation revealed elevated D-dimer levels and mild leukocytosis, while other baseline parameters were within normal limits. The patient’s laboratory parameters are summarized in Table 1.

**Table 1:** Summary of laboratory investigations with reference ranges

Parameter	Value	Reference range
Hemoglobin	14.8 g/dL	13-17 g/dL
Total leukocyte count	12,000/µL	4,000-11,000/µL
Platelet Count	200,000/µL	150,000-450,000/µL
D-dimer	3140 ng/mL	<500 ng/mL
aPTT	36 seconds	25-35 seconds
Homocysteine	98 µmol/L	5-15 µmol/L

A chest X-ray showed left hilar infiltration, and electrocardiography displayed the S1Q3T3 pattern. Considering the possibility of pulmonary thromboembolism, the patient was thrombolyzed with streptokinase (250,000 IU over 1 hour and 100,000 IU/hr for 12 hours). Lower limb venous Doppler ultrasonography showed no evidence of deep vein thrombosis. The hypercoagulable workup, including protein C, S, antithrombin III, and the factor V Leiden gene mutation, was normal. Thus, further workup for antiphospholipid antibodies was ordered, which was also negative. Ophthalmologic evaluation revealed bilateral posterior subcapsular cataracts.

**Figure 1:**
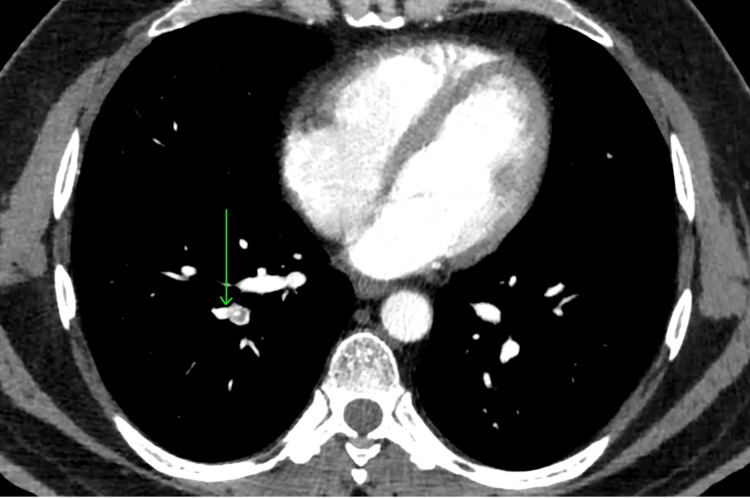
Axial CT pulmonary angiography showing partial hypodense filling defects in segmental and subsegmental branches of the pulmonary arteries, consistent with pulmonary thromboembolism.

Considering the combination of unexplained thromboembolism and early-onset cataract, plasma homocysteine levels were assessed and found to be markedly elevated at 98 µmol/L. This was found to be consistent with intermediate-to-severe HHcy. CT pulmonary angiography (CTPA) confirmed massive pulmonary embolism, demonstrating partial hypodense filling defects in segmental and subsegmental branches (Figure 1).

The patient was stabilized and discharged with oral anticoagulation using dabigatran (150 mg twice daily). Given the presence of HHcy, a recognized prothrombotic condition, extended anticoagulation was considered. Concurrent vitamin supplementation with folic acid, pyridoxine, and vitamin B12 was initiated to address the underlying metabolic abnormality. He was referred for cataract surgery too.

## Discussion

HHcy is a metabolic disorder characterized by elevated plasma homocysteine. This is a sulfur-containing amino acid produced during methionine metabolism. Homocysteine is normally metabolized through two pathways: remethylation to methionine, which needs folate and vitamin B12, and trans-sulfuration to cysteine, which needs vitamin B6. Disruption of any of these pathways leads to the accumulation of homocysteine in circulation. Elevated homocysteine has been widely recognized as a risk factor for vascular pathologies due to its association with endothelial dysfunction, oxidative stress, and prothrombotic activity [1, 2].

HHcy can cause venous thromboembolism through many mechanisms, such as endothelial injury, increased oxidative stress, decreased nitric oxide availability, and the activation of the coagulation cascade [3, 8].Studies suggest that elevated homocysteine levels can increase the risk of venous thromboembolism by up to 2.5-fold [9]. In our case, too, HHcy is the most plausible contributor to pulmonary embolism since other thrombophilia conditions were excluded.

The ophthalmic manifestations of HHcy are getting more recognition. Mainly, these conditions include retinal vascular occlusions, glaucoma, optic neuropathy, and cataract formation [4].Out of these, cataracts are implicated to occur via many metabolic and oxidative mechanisms affecting lens homeostasis. Structural stability of crystalline proteins and maintenance of a balanced redox environment determine lens transparency. As homocysteine contains a reactive sulfhydryl group, which can generate reactive oxygen species (ROS), it overwhelms antioxidant defenses and induces oxidative damage to lens proteins [5, 6]. This oxidative stress leads to abnormal disulfide bonding and aggregation of crystallins, which further results in progressive lens opacification [6, 7].

Experimental studies have shown that homocysteine leads to endoplasmic reticulum (ER) stress in the epithelial cells of the lens. ER stress then activates the unfolded protein response, leading to impaired protein folding and promoting the accumulation of misfolded crystallins. Further, HHcy may also interfere with autophagy. Thus, oxidative stress, ER stress, and impaired protein clearance together hasten the process of cataract formation [9]. Homocysteine-induced mitochondrial dysfunction also triggers apoptosis of lens epithelial cells. The loss and abnormal migration of these cells toward the posterior capsule lead to the formation of posterior subcapsular cataracts. This commonly manifests in patients as glare, reduced visual acuity, and difficulty in near vision [10]. Since the posterior subcapsular region is a metabolically active region, it is susceptible to oxidative and metabolic damage.

Cataracts are usually an age-related condition. But they can occur at any stage of life and in different forms, like congenital, juvenile, and early-onset. Cataracts that develop before the age of 45-50 years are categorized as early-onset types and frequently have an identifiable metabolic or systemic cause [11]. In the younger population, cataracts may arise secondary to conditions like diabetes mellitus, ocular trauma, chronic corticosteroid therapy, high myopia, or metabolic disorders such as HHcy [11, 12]. Due to the absence of these other factors in our patient, HHcy is the most likely factor for cataract development. Population-based studies support this association too. The Blue Mountains Eye Study showed that higher serum homocysteine concentrations were associated with an increased prevalence of posterior subcapsular and cortical cataracts [13]. People with homocysteine levels greater than 15 µmol/L have more than two times the risk of developing cortical cataracts compared to people of normal levels [13]. Similar findings have been reported in other studies where higher homocysteine levels were seen in cataract patients over healthy controls [14].

Clinical data have also suggested the threshold levels associated with cataract risk. Plasma homocysteine concentrations below 10 µmol/L are considered nearly normal. Levels that are over 15 µmol/L are defined as HHcy. Moderate elevations (>30 µmol/L) are the ones that are mostly seen in metabolic disorders. Extremely high levels (>100 µmol/L) are strongly associated with early-onset cataracts and ocular abnormalities such as ectopia lentis [15]. Management of HHcy focuses on correcting the underlying metabolic abnormality. Supplementation with folic acid, vitamin B12, and vitamin B6 remains the main therapy, as these vitamins act as cofactors in homocysteine metabolism [16]. Riboflavin may also further improve homocysteine metabolism in individuals with MTHFR polymorphisms [17]. Even though vitamin supplementations lower homocysteine levels, they have not consistently been shown to reduce recurrent thromboembolic events [16].

## Conclusions

This case highlights HHcy as a potential metabolic link between systemic vascular disease and early-onset posterior subcapsular cataract. Recognizing this association is important as patients who present with unexplained early cataract can benefit from metabolic evaluation for HHcy, to start targeted therapy and timely ophthalmologic management.
